# Efficacy of Mineral Trioxide Aggregate Versus Biodentine as a Direct Pulp Capping Material in Carious Human Mature Permanent Teeth: A Systematic Review

**DOI:** 10.7759/cureus.89154

**Published:** 2025-07-31

**Authors:** Rashmi Misra, Nikita Toprani, Sumita Bhagwat, Aashaka Vaishnav, Aastha Dureja, Omkar Bhosale

**Affiliations:** 1 Department of Conservative Dentistry and Endodontics, DY Patil School of Dentistry, Navi Mumbai, IND

**Keywords:** biodentine, carious teeth, clinical success, dentin bridge formation, direct pulp capping, mature permanent molars, mineral trioxide aggregate

## Abstract

The present systematic review and meta-analysis aimed to evaluate and compare the clinical efficacy of Mineral Trioxide Aggregate (MTA) and Biodentine (BD) as direct pulp capping materials in cariously exposed mature permanent teeth, focusing on outcomes such as pulp vitality preservation, dentin bridge formation, and complication rates, including tooth discoloration. A comprehensive literature search was conducted across PubMed, Scopus, Web of Science, and other dental databases following Preferred Reporting Items for Systematic Reviews and Meta-Analyses (PRISMA) guidelines. The review protocol was registered in PROSPERO (CRD42023463513). Eligible studies included randomized controlled trials and retrospective studies reporting clinical and radiographic success. Risk of bias was assessed using the Cochrane Risk of Bias Tool and the Newcastle-Ottawa Scale. Eleven studies involving 373 treated teeth were included. Meta-analysis revealed a pooled risk ratio (RR) for success of 0.98 (95% CI: 0.94-1.03), slightly favoring MTA but without statistical significance. BD showed a lower failure rate (RR = 1.94; 95% CI: 0.48-7.94) and marginally better outcomes in calcific bridge formation (RR = 0.90; 95% CI: 0.75-1.08), though these differences were also not statistically significant. Biodentine demonstrated superior handling, a shorter setting time (about 12 minutes), and no risk of discoloration, which is an issue noted with MTA. Both materials are clinically effective, but BD may be preferable due to its practical and aesthetic advantages. Further long-term, standardized studies are recommended.

## Introduction and background

Direct pulp capping (DPC) is a conservative procedure aimed at protecting vital pulp that has been exposed by caries or trauma, thereby postponing or eliminating the need for root-canal therapy [1]. Success depends greatly on the material applied directly over the pulp wound. The material should seal the exposure, encourage reparative dentin, and remain stable in the oral environment. Over the past three decades, calcium silicate-based materials have been favored by dental clinicians, among which Mineral Trioxide Aggregate (MTA) and Biodentine (BD) have attracted particular clinical attention [2]. Comparing their performance in mature permanent teeth affected by carious lesions has become relevant because clinicians often base their choice on handling convenience or anecdotal preference rather than a balanced interpretation of evidence.

MTA was introduced in the mid-1990s and quickly earned a reputation for excellent biocompatibility and sealing ability. Its high pH, release of calcium ions, and capacity to form hydroxyapatite at the dentin interface promote predictable dentin bridge formation, making it the material against which newer products are measured [3]. Despite these advantages, MTA has certain drawbacks, which include a long setting time, potential for discoloration, and difficult manipulation. These limitations reduce the routine use of MTA in busy clinical settings where chairside time is generally limited. These practical issues have motivated researchers and manufacturers to look for alternatives that maintain biological benefits while offering better handling.

BD is another tricalcium silicate cement that has been more recently introduced. It incorporates additives that shorten the setting time and improve consistency, thereby overcoming the drawbacks associated with MTA. Laboratory studies suggest BD releases higher early levels of calcium and silicate ions, which can stimulate odontoblast-like cell differentiation and speed up mineral deposition [4]. Clinically, BD is supplied as a ready-to-mix capsule, reducing operator variability and chairside time. Its light ivory color may also lessen the risk of coronal staining. The prospect of achieving outcomes comparable to MTA with simpler manipulation has sparked widespread interest and prompted a growing number of comparative clinical trials.

Both MTA and BD promote healing of the pulp-dentin complex primarily through the release of calcium ions, which react with phosphate ions in tissue fluids to form hydroxyapatite [3,4]. This biomineralization process not only seals the pulp exposure but also promotes the differentiation of pulpal stem cells into odontoblast-like cells, thereby stimulating reparative dentin formation. The high alkalinity of MTA contributes to its antimicrobial properties and creates an environment conducive to tissue regeneration [3]. Similarly, BD induces the formation of a calcium phosphate layer on its surface, which enhances its bioactivity. Its smaller particle size and optimized formulation result in faster ion release and early mineralization, making it particularly effective in triggering odontogenic cell activity and dentin bridge formation [4].

Although individual studies have reported high success with both cements, their designs vary considerably in terms of case selection, pulpal status assessment, operative technique, and follow-up duration [2]. These differences make it difficult to draw firm conclusions from any single report. A systematic review of available evidence is, therefore, needed to clarify whether BD truly matches or surpasses MTA in maintaining pulp vitality in carious mature teeth.

The present review addresses this gap by evaluating clinical and histological outcomes, success and survival rates, and factors that might influence performance. The objective of the review is to provide Indian clinicians and researchers with a clear, evidence-based perspective on the relative merits of MTA and BD for DPC. It would guide practitioners who must choose a material that balances biological success, ease of use, cost, and patient acceptance.

## Review

Methdology

Research Design

The present systematic review was conducted to compare the efficacy of MTA and BD as DPC materials in carious mature permanent teeth. The entire review process was guided by the Preferred Reporting Items for Systematic Reviews and Meta-Analyses (PRISMA) guidelines [5]. The review protocol was registered in the PROSPERO database (Reference ID: CRD42023463513).

Research Question

The central research question guiding this systematic review was: What is the comparative efficacy of MTA and BD as direct pulp capping agents in mature permanent teeth with carious pulp exposures, based on clinical and radiographic outcomes? The review aimed to address differences in pulp vitality preservation, dentin bridge formation, postoperative symptoms, and long-term treatment success between the two materials.

Search Strategy

A comprehensive and systematic electronic search was performed across multiple databases, including PubMed, Scopus, Web of Science, and the Cochrane Library. The search incorporated both Medical Subject Headings (MeSH) and free-text terms relevant to the topic. Keywords used in combination included: “Mineral Trioxide Aggregate,” “MTA,” “Biodentine,” “direct pulp capping,” “carious exposure,” “mature permanent teeth,” and “clinical success.” Boolean operators such as “AND” and “OR” were applied to refine the search results. The search was restricted to articles published in English and indexed from the inception of each database up to the final date of the search in December 2024. Reference lists of relevant studies and previous reviews were also manually scanned to identify any additional eligible articles.

Eligibility Criteria

Studies were included if they directly compared MTA and BD as DPC materials in human mature permanent teeth with carious exposures. Eligible studies included randomized controlled trials, prospective and retrospective cohort studies, and clinical comparative studies reporting on at least one of the following outcomes: clinical success (absence of symptoms, vitality maintenance), radiographic findings (periapical status, dentin bridge), or patient-reported outcomes. Only peer-reviewed full-text articles available in English were considered. Exclusion criteria included in vitro studies, animal studies, case reports, review articles, conference abstracts, and studies not involving carious exposures. The PICOS (Population, Intervention, Comparison, Outcomes, and Study) criteria used for determining the eligibility of the articles are listed in Table 1.

**Table 1:** PICOS Framework for the present systematic review PICOS: Population, Intervention, Comparison, Outcomes and Study; MTA: Mineral Trioxide Aggregate; BD: Biodentine; DPC: Direct Pulp Capping.

PICOS Component	Inclusion Criteria	Exclusion Criteria
Population (P)	Human participants with carious pulp exposure in mature permanent teeth (closed apex)	Studies on primary teeth, immature permanent teeth, animal or in vitro studies
Intervention (I)	Direct pulp capping performed using MTA	Studies using MTA for indirect pulp capping or pulpotomy, not DPC
Comparator (C)	Direct pulp capping performed using BD	Studies comparing MTA with materials other than BD (e.g., calcium hydroxide, CEM cement, Theracal)
Outcomes (O)	Clinical success (pulp vitality, absence of symptoms), radiographic success (no periapical pathology), dentin bridge formation, discoloration, failure rates	Studies not reporting any of the primary or secondary outcomes related to pulp vitality or treatment success
Study Design (S)	Randomized controlled trials (parallel-arm or split-mouth), retrospective comparative clinical studies	Case reports, case series, narrative reviews, editorials, letters, conference abstracts, in vitro and animal studies

Study Selection Process

Two reviewers independently screened all titles and abstracts obtained from the electronic search to identify potentially relevant studies. Full texts were retrieved for those meeting the inclusion criteria or where eligibility was unclear. The selected full-text articles were then evaluated in detail against the eligibility criteria. Disagreements between the reviewers were resolved through discussion, and if needed, a third reviewer was consulted to achieve consensus.

Data Extraction

A structured data extraction form was developed and used to collect key information from each included study. The extracted data included study details (author, publication year, country), study design, sample size, age group of participants, material used (MTA or BD), clinical procedures followed, follow-up duration, outcome measures assessed, and results reported. Clinical and radiographic success, adverse events, dentin bridge formation, and patient-related outcomes were noted wherever available.

Assessment of Study Quality and Risk of Bias

The quality of non-randomized studies was evaluated using the Newcastle-Ottawa Scale, which assesses methodological quality based on three domains: selection of participants, comparability of study groups, and outcome/exposure assessment [6]. Each study was assigned a score out of nine. Studies scoring 7-9 were considered high quality, 4-6 moderate quality, and 0-3 low quality. For randomized controlled trials, the Cochrane Risk of Bias (RoB-2) tool was employed [7]. This tool evaluates potential bias across several domains, such as randomization, allocation concealment, blinding, outcome data completeness, and selective reporting. Visual summaries of the risk of bias across studies were generated using Review Manager (RevMan) version 5.3.

Data Synthesis

The findings of the included studies were qualitatively synthesized to compare the effectiveness of MTA and BD in terms of preserving pulp vitality, promoting dentin bridge formation, and ensuring long-term tooth survival. Where study designs and reported outcomes were sufficiently homogeneous, a meta-analysis was planned. For outcomes that varied greatly in terms of definitions, measurement methods, or follow-up durations, a narrative synthesis approach was followed to highlight consistent patterns, discrepancies, and clinically relevant findings. The goal of synthesis was to present a balanced and evidence-based understanding of how both materials perform in the clinical setting.

Results

**Figure 1:**
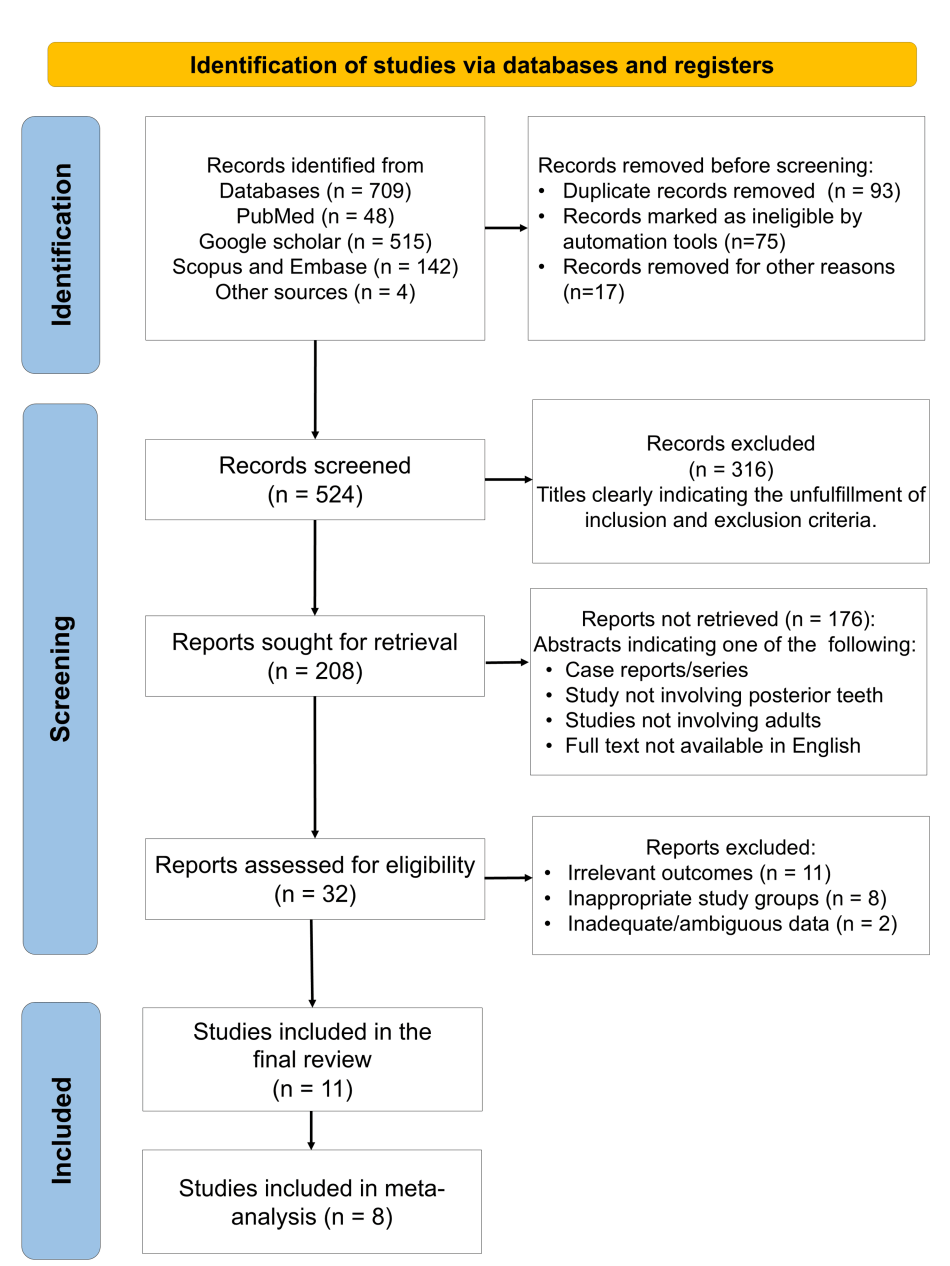
PRISMA flow diagram indicating the selection process of the articles for data synthesis in the present systematic review PRISMA: Preferred Reporting Items for Systematic Reviews and Meta-Analyses.

A total of 11 studies published from the year 2016 to 2022 were identified [8-18]. The study selection process is depicted in Figure 1.

The data extracted from these studies related to study characteristics are comprehensively summarized in Table 2, and those related to procedures and outcomes are summarized in Table 3.

**Table 2:** Data related to characteristics of the studies included in the present systematic review RCT: randomized controlled trial, BD: Biodentine, MTA: mineral trioxide aggregate, wMTA: white mineral trioxide aggregate, gMTA: gray mineral trioxide aggregate, CaOH: calcium hydroxide, CEM: calcium-enriched mixture, NP: not provided, PDL: periodontal ligament, SSC: stainless steel crown.

Sr. No.	Author	Year	Country	Sample size	Age group	Study design	Inclusion Criteria	Radiographic inclusion criteria	Exclusion criteria	Groups
1	Hegde et al. (2016) [8]	2016	India	24	18 to 40	RCT	• Deep dental caries in molars • Asymptomatic, responded positively to thermal and electrical tests with no tenderness on percussion	• No pathologic changes on periapical radiographs	• Thickened periodontal ligament • Furcation radiolucencies • Periradicular pathosis	BD and wMTA
2	Brizuela et al. (2017) [9]	2017	Spain	169	7 to 16 (11.3)	RCT	• Less than 2 mm of carious exposure in a permanent molar • Pulpal testing compatible with normal pulp or reversible pulpitis	NP	• Systemic and/or neurologic pathology • Teeth with radiologic signs of internal resorption or pulpal calcifications, • No restorable teeth • Uncontrollable pulpal bleeding.	CaOH, BD, and wMTA
3	Katge and Patil (2017) [10]	2017	India	100	7 to 9	Split-mouth RCT	• Bilateral asymptomatic first permanent molars with carious involvement • Pinpoint inadvertent pulp exposure during caries excavation • Bleeding controlled under pressure at the exposure site and vital pulp.	• Radiolucency involving enamel, dentin, and approaching the pulp	• Unilateral carious first permanent molars • Uncontrollable bleeding • Non-vital pulp, large pulp exposure (more than 1 mm) • Presence of spontaneous pulpal pain, intraoral or extraoral swelling, sinus tract formation, and carious pulpal exposure. • Pulp calcification, internal or external root resorption, and periapical radiolucency	BD and gMTA
4	Linu et al. (2017) [11]	2017	India	26	15 to 30	Retrospective study	• Age 15 to 30 years • Complaints of cavity in mature permanent teeth and/or sensitivity to cold food and/or food lodgement in cavity at presentation • No history of night pain or spontaneous pain • Mandibular molars with caries restricted to occlusal surface • Pulp sensibility tests elicited a positive response • Patients who were systemically healthy	• Radiographic examination showed deep caries approaching pulp, with no signs of periapical pathology	• Teeth with excruciating/lingering pain in response to pulp sensibility tests and iatrogenic pulp exposure	BD and MTA
5	Parinyaprom et al. [12]	2017	Thailand	59	6 to 18	RCT	• Permanent tooth with deep caries • Positive response to cold test; • No associated swelling, pus exudate, fistula, or abnormal mobility • Pulp exposure that was not larger than 2.5 mm in diameter • Vital pulp, • Pulpal bleeding controlled within 10 minutes • The tooth could be restored with resin composite, amalgam, or stainless steel crown (SSC).	• Presence of caries radiolucency penetrating into three-fourths or more of the entire dentin thickness • No prominent radiolucency in the furcation or periapical regions, internal or pathologic external root resorption, or calcification/or pulp canal obliteration.	*Early periapical lesions, such as widened PDL space or condensing osteitis, were not considered an exclusion criterion for this study.	BD and MTA
6	Awawdeh et al. (2018) [13]	2018	Jordan	68	16 to 51 (32.5)	RCT	• Good general health • Carious lesion penetrating more than half the thickness or more into dentin and involving two walls, • Severe symptoms but diagnosis indicating reversible pulpitis based on the cold test • Complaint of tooth pain	Considered, but the exact criteria not mentioned	• Immature teeth, unrestorable teeth, unresponsive to thermal stimulation or electric pulp testing, • Presence of sinus tracts or swelling, periapical rarefaction • Any serious medical problem that prevented the patient from receiving treatment or attending follow-up visits.	wMTA and BD
7	Paula et al. (2019) [14]	2019	Portugal	21	18 to 55	retrospective clinical study	• Thermal sensitivity response compatible with vital tooth • Age 18 to 55 years • Reasonable state of health and oral hygiene • Without periodontal pathology	NP	NP	wMTA and BD
8	Hoseinifar et al. (2020) [15]	2020	Iran	40	14 to 25	RCT	• Human first and second maxillary and mandibular premolar teeth • Without restoration, periodontal disease and caries, with healthy pulp • Age 14-25 years scheduled for extraction for orthodontic treatment	NP	NP	BD, calcium-enriched mixture (CEM), and MTA
9	Ahlawat et al. (2022) [16]	2022	India	142	NP	RCT	• Maxillary and mandibular first and second molars • Deep carious lesions		NP	BD and wMTA
10	Anwar et al. (2022) [17]	2022	Pakistan	150	18 to 45	RCT	• Maxillary and Mandibular permanent teeth. • Deep caries • No prior restoration		NP	MTA and BD
11	Krishnamurthy and Raju (2022) [18]	2022	India	24	19 to 40	RCT	• Patients between 19 and 40 years of age. • Carious exposure less than two millimetres in a molar. • Pulpal response on diagnostic tests should be with an adjunct to a healthy tooth or pulp in reversible pulpitis	• Teeth not having signs and symptoms related to calcifications in pulp or internal resorption	• Patients with systemic diseases. • Restoration contraindicated or not possible for the tooth. • Uncontrolled and excessive bleeding in the pulpal exposure site.	Dycal, MTA, and BD

**Table 3:** Data related to outcomes of the studies included in the present systematic review CHX: chlorhexidine, LA: local anesthesia, NaOCl: sodium hypochlorite, DPC: direct pulp capping, MTA: mineral trioxide aggregate, wMTA: white mineral trioxide aggregate, BD: Biodentine, RMGIC: resin-modified glass ionomer cement, GIC: glass ionomer cement, SSC: stainless steel crown, CEM: calcium enriched mixture, NP: not provided.

Sr. No.	Author	Cleaning/disinfection	LA	Isolation	Homeostasis at the exposure site	MTA	Biodentin	Restoration	Follow-up	Conclusive indings
1	Hegde et al. (2016) [8]	• 0.2% CHX solution.	1:100,000 lignocaine hydrochloride with adrenaline	Rubber dam	3% NaOCl	2 mm thick layer of ProRoot WMTA on the exposed pulp and surrounding dentin, zinc polycarboxylate temporary	Filled cavity and left as a temporary restoration	RMGIC and composite resin	3 mo, 6 mo, 12 mo	• MTA and BD are reliable DPC agents. • Careful case selection, isolation, complete caries excavation, pulp capping, and proper restoration will contribute to the success of the treatment
2	Brizuela et al. (2017) [9]	• 0.2% CHX solution.	2% lidocaine hydrochloride with epinephrine 1:80,000	Rubber dam	Pressure with cotton pellets soaked in Saline	2 mm thick layer of WMTA was applied over the pulp with a gun system and a wet cotton pellet until the exposed pulp was completely covered.	2 mm thick layer was applied over the pulp with a gun system and a wet cotton pellet until the exposed pulp was completely covered.	Composite resin	1 wk, 3 mo, 6 mo, 1 year	There were no statistically significant differences among the materials studied for pulp capping procedures in carious teeth of children. All showed high success rates during a 1-year follow-up period. MTA and BD are viable alternatives to CH for pulp capping procedures. BD showed 100% success, has the advantage of easy handling, sets in approximately 12 minutes, and does not cause discoloration of the tooth
3	Katge and Patil (2017) [10]	• Pumice • Rubber cup	2% lidocaine	Rubber dam	3% NaOCl	A layer of MTA followed by RMGIC	Filled cavity and left as a temporary restoration, later reduced to a 1 mm layer	RMGIC over MTA and composite resin	6 and 12 mo	• 100% success rate with both MTA and BD when used as a DPC agent in first permanent molars in 7- to 9-year-old children. • Clinically and radiographically, there was no significant difference between MTA and BD as a DPC agent in young permanent molars. • After a 6-month follow-up period, the number of teeth showing dentin bridge formation was higher in the MTA group as compared with the BD group, but these findings were statistically nonsignificant. • After a 12-month follow-up, the number of teeth showing dentin bridge formation was higher in the BD group when compared with the MTA group.
4	Linu et al. (2017) [11]	• Pumice slurry • Rubber cup, • 5% NaOCl	Lignocaine with 2%, adrenaline 1:200000;	Rubber dam	5% NaOCl	Placed over the exposure site and surrounding dentin as a 1.5- to 3.0-mm-thick layer	Placed over the exposure site and then bulk filled	RMGIC over MTA	1, 3, 6, 12, and 18 mo	The MTA group showed a success rate of 84.6% (11/13) and the BD group a rate of 92.3%
5	Parinyaprom et al. [12]	2.5% NaOCl	4% articaine with 1:100,000 epinephrine	Rubber dam	2.5% NaOCl	1.5 mm thickness of ProRoot MTA was placed on the pulp exposure site and surrounding dentin. Then, RMGIC was placed immediately over the ProRoot MTA as a base material	BD was placed as a pulp dressing as well as a base material and allowed to set, usually in 12 minutes	All teeth were restored with resin composite, amalgam, or an SSC, depending on the amount of	6, 10, 18, 18-54 mo (every 6 mo)	• Success rate 92.6% with ProRoot MTA and 96.4% with BD • Survival probabilities of DPC with ProRoot MTA and BD were 0.92 and 0.96 • Noticeable gray discoloration was observed only with ProRoot MTA (55%) • Compared with ProRoot MTA, BD resulted in a non-inferior success rate when used as a DPC material. • BD did not cause any gray discoloration in this study and may be recommended for DPC in the esthetic zone
6	Awawdeh et al. (2018) [13]	CHX	lidocaine and epinephrine (1:100,000)	Rubber dam	5% NaOCl	Filled half the cavity. Then, temporized with Intermediate Restorative Material placed over a piece of moistened cotton	Filled half the cavity. Then, temporized with Intermediate Restorative Material placed over a piece of moistened cotton	Amalgam or a resin composite	6 mo, 1, 2, and 3 yr	• No significant differences in success rates between BD- and wMTA-treated teeth • All wMTA teeth exhibited some evidence of discoloration, whereas there was no noticeable discoloration in the BD group.
7	Paula et al. (2019) [14]	2% CHX	NP	Rubber dam	NP	WhiteProRoot® MTA followed by placement of GIC	WhiteProRoot® MTA followed by placement of GIC	Light-cured composite resins	1w, 1 mo, 3 mo, 6 mo	• Success rates of tricalcium silicate cement were 95%, which are high and similar to the 100% success rate of MTA-based cement. • Comparisons showed that the success rates obtained are not dependent on age or tooth type, or on the cavity or aetiology of pulp exposure.
8	Hoseinifar et al. (2020) [15]	• Rubber cup • Prophylaxis paste • 0.2% CHX	2% lidocaine	Rubber dam	NP	NP	NP	Resin-modified glass ionomer	NP	• The dentin bridge formation and the thickness of dentin bridge formed in the BD group were higher than in the other groups • Pulp showed greater inflammation compared to CEM cement and MTA. • The results of this study suggested that MTA and CEM cement performed better when employed as the DPC material.
9	Ahlawat et al. (2022) [16]	NP	NP	NP	NP	Placed over the pulp exposure spot. After 10 min, the cavit was placed over the MTA	Placed over the exposure spot until completely packed.	Composite resin	3, 6, 9, and 12 mo	• The success rate for MTA was 94% and BD was 100% (not significant • Direct pulp capping with MTA and BD, after pulp exposure during excavation of deep caries in reversible pulpitis, is a successful treatment option that was able to maintain pulp vitality in permanent teeth at the end of a 1‑year follow‑up
10	Anwar et al. (2022) [17]	0.2% CHX	NP	Rubber dam	5.25% NaOCl	A 2-mm-thick layer of cement on the exposed site. Damp cotton placed on the tooth was initially restored using temporary material (CAVIT)	The whole cavity was filled with BD	Amalgam or a resin composite	3 mo	• BD is more effective than MTA. • It can be used as a bulk fill, simplifying the pulp capping procedure • BD is preferred owing to its ease of placement and good setting time
11	Krishnamurthy and Raju (2022) [18]	2% CHX	2% Lidocaine hydrochloride with epinephrine 1:80,000	Rubber dam and saliva ejector	2% Lidocaine with adrenaline	Placed with MTA carrier in the floor of the cavity approximately 2*2 mm over the pulp exposed area.	Placed approximately 2*2 mm over the pulp exposed area.	GIC	1, 3, 6 mo	• BD is better than MTA for direct pulp capping.

Out of the included 11 studies, the majority (n = 5) were conducted in India, while other studies were distributed across various countries, including Spain, Thailand, Jordan, Portugal, Iran, and Pakistan. The majority of the studies (n = 9) were randomized clinical trials (RCTs), one of which had a split-mouth design [10], while the others had a parallel-arm design. Two studies compared the efficacy of MTA and BD as pulp capping materials retrospectively through archival records. It was ensured that the protocols followed for the placement of the respective materials were maintained throughout the years for the findings of the study to be reliable and valid. The sample size across studies varied from 24 to 169. The age groups of study populations were around 18 to 45 years in six studies, and six to 18 years in three studies.

The studies used inclusion criteria such as the presence of deep dental caries in molars, asymptomatic teeth that responded positively to thermal and electrical tests with no tenderness on percussion, less than 2 mm of carious exposure in a permanent molar, pulpal testing compatible with normal pulp or reversible pulpitis, pinpoint inadvertent pulp exposure during caries excavation, bleeding controlled under pressure at the exposure site, vital pulp, complaints of the cavity in mature permanent teeth and/or sensitivity to cold food and/or food lodgment in the cavity at presentation, no history of night pain or spontaneous pain, mandibular molars with caries restricted to the occlusal surface, pulp sensibility tests eliciting a positive response, and patients who were systemically healthy. The study with a split-mouth design also included bilateral asymptomatic first permanent molars with carious involvement [10]. Six studies used radiographic criteria along with the clinical ones, which included radiographic examination showing deep caries approaching the pulp, with no signs of periapical pathology.

The various exclusion criteria used across different studies were the presence of unilateral carious first permanent molars, uncontrollable bleeding, non-vital pulp, large pulp exposure (more than 1 mm), presence of spontaneous pulpal pain, intraoral or extraoral swelling, sinus tract formation, carious pulpal exposure, pulp calcification, internal or external root resorption, and periapical radiolucency. Five studies mentioned the use of white MTA, and one study used gray MTA. Other studies (n = 5) did not specify the type of MTA used.

Most of the studies (n = 7) used chlorhexidine for cleaning and sterilization of the oral cavity or the environment of the tooth before the commencement of the pulp capping procedure. The concentration of chlorhexidine used varied from 0.2% to 2% across different studies. Three studies performed prophylaxis with pumice and rubber cups, while two studies used sodium hypochlorite in concentrations of 2.5% and 5%, respectively. All the studies used a rubber dam for isolation of the tooth, with additional cotton rolls and a saliva ejector if needed.

Since the DPC procedure involves pinpoint exposure of the pulp chamber, the procedure is usually painful and requires the administration of local anesthesia. In seven studies, 2% lidocaine with 1:80,000 to 1:200,000 adrenaline was used as the local anesthetic agent. Parinyaprom et al. (2017) employed 4% articaine with 1:100,000 as the local anesthetic agent in their study [12].

In the DPC procedure, the blood oozing from the pulp exposure needs to be stopped before the capping agent can be applied, or else the material is unable to set normally and may get dislodged. To achieve hemostasis, six studies used cotton pellets soaked with sodium hypochlorite in a concentration of 2.5% to 5%, and one study by Krishnamurthy and Raju (2022) used 2% lidocaine with adrenaline [18]. Brizuela et al. (2017) used only pressure from the cotton pellet without any agent to stop the pulpal bleeding [9].

As for the protocol for the application of MTA for DPC, the majority of the investigators applied a 1.5 to 3 mm thick layer of MTA, followed by temporization with cotton and cement to fill the cavity. BD, on the other hand, was unanimously used as the pulp capping agent as well as the bulk-filling interim restorative material. The final restoration was mostly performed using RMGIC and composite resin, except for three studies, which also used amalgam.

The follow-up periods across the studies ranged from 1 week to 18 months, with the follow-up period of three to six months being common across all the studies. Most of the studies found that although the success rate of teeth treated with BD was slightly higher than those treated with MTA, there were no significant differences. However, the teeth treated with white MTA exhibited some evidence of discoloration, whereas there was no noticeable discoloration in the BD group. Hoseinifar et al. (2020) found that the dentin bridge formation and the thickness of the dentin bridge formed in the BD group were higher than in the other groups, while the pulp showed greater inflammation compared to CEM cement and MTA [15]. The conclusive findings of the studies included in the present systematic review suggested that BD is more advantageous compared to MTA when employed as the DPC material.

Methodological Quality of Included Studies

**Figure 2:**
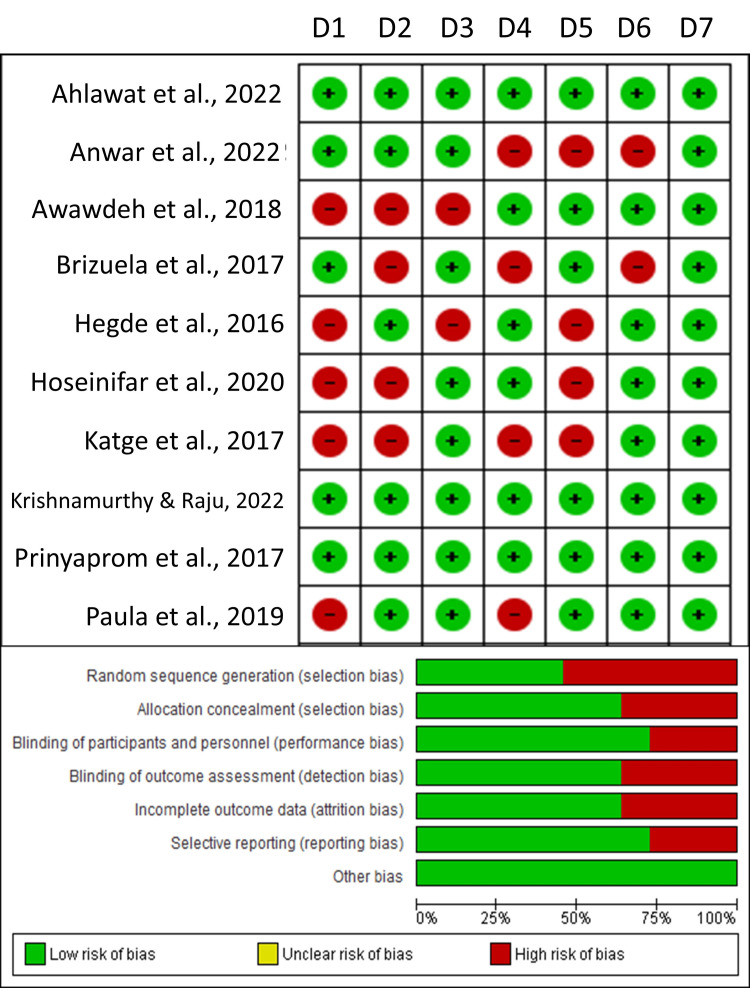
Risk of bias across clinical studies included in the present systematic review Data from [8-10, 12-18].

The included studies had variable ratings across the various domains of risk of bias (Figure 2). The highest risk of bias was seen for random sequence generation (selection bias), followed by blinding of outcome assessment (detection bias), incomplete outcome data (attrition bias), and selective reporting (reporting bias). Among the included studies, Ahlawat et al. (2022), Krishnamurthy and Raju (2022), and Parinyaprom et al. (2017) reported the lowest risk of bias among all studies, while the remaining studies reported moderate to lowest risk of bias with regard to other domains [12,16,18]. Domains of allocation concealment (selection bias), blinding of participants and personnel (performance bias), and other biases were given the lowest risk of bias by the included studies.

The sole retrospective study included in the review, Linu et al. (2017), was appraised with the Newcastle-Ottawa Scale and received an overall quality score of 6 out of 9, indicating moderate methodological quality [11]. Specifically, it earned two of the four possible stars in the “Selection” domain, demonstrating partially adequate case definition and representativeness; it secured the full two stars available for “Comparability,” reflecting appropriate control for key confounders; and it obtained two of the three stars in the “Exposure” domain, signifying acceptable ascertainment of exposure and follow-up procedures.

Synthesis of results

The risk ratio (RR) is used as a summary statistic measure for dichotomous outcomes. The outcomes were assessed in terms of better efficacy between MTA and BD in terms of success rate, failure rate, and calcific bridge formation.

Success Rate

**Figure 3:**
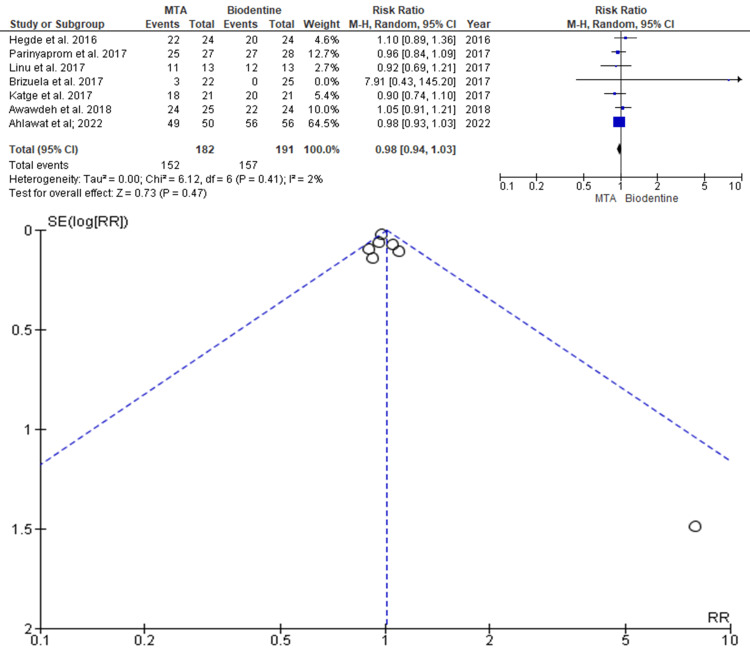
Forest plot showing comparison between Mineral Trioxide Aggregate and Biodentine with regard to better success rate and funnel plot showing asymmetric distribution with absence of systematic heterogeneity Data from [8-13,16].

Data was evaluated from seven studies from an aggregate of 373 teeth, of which 182 teeth were evaluated by MTA and 191 teeth were evaluated by BD for the evaluation of the better efficacy between the two modalities in terms of better success rate as an outcome. As shown in Figure 3, the RR is 0.98 (0.94-1.03), and the pooled estimates favour MTA. This signifies that a better success rate on average was 0.98 times more in the MTA group compared to the BD group, and this difference is not statistically significant (p>0.05). The funnel plot did not show significant asymmetry, indicating the absence of publication bias.

Failure Rate

**Figure 4:**
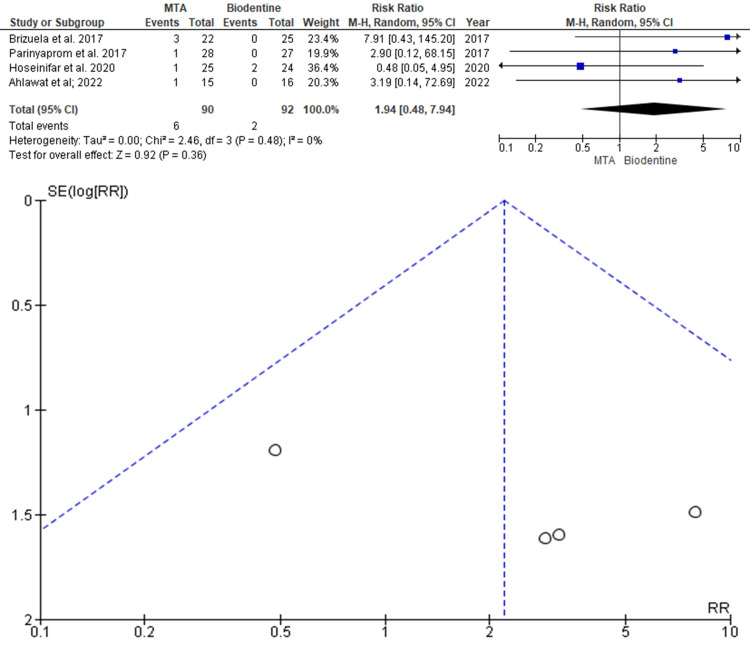
Forest plot showing the comparison between Mineral Trioxide Aggregate and Biodentine with regards to the failure rate and funnel plot showing asymmetric distribution with the presence of systematic heterogeneity Data from [9,12,15,16].

Data was evaluated from four studies from an aggregate of 182 teeth, of which 90 teeth were evaluated by MTA and 92 teeth were evaluated by BD for the evaluation of the better efficacy between the two modalities in terms of failure rate as an outcome. As shown in Figure 4, the RR is 1.94 (0.48-7.94), and the pooled estimates favour BD. This signifies that the failure rate on average was 1.94 times higher in the BD group compared to the MTA group, and this difference is not statistically significant (p>0.05). The funnel plot did show significant asymmetry, indicating the presence of publication bias.

Calcific Bridge Formation

**Figure 5:**
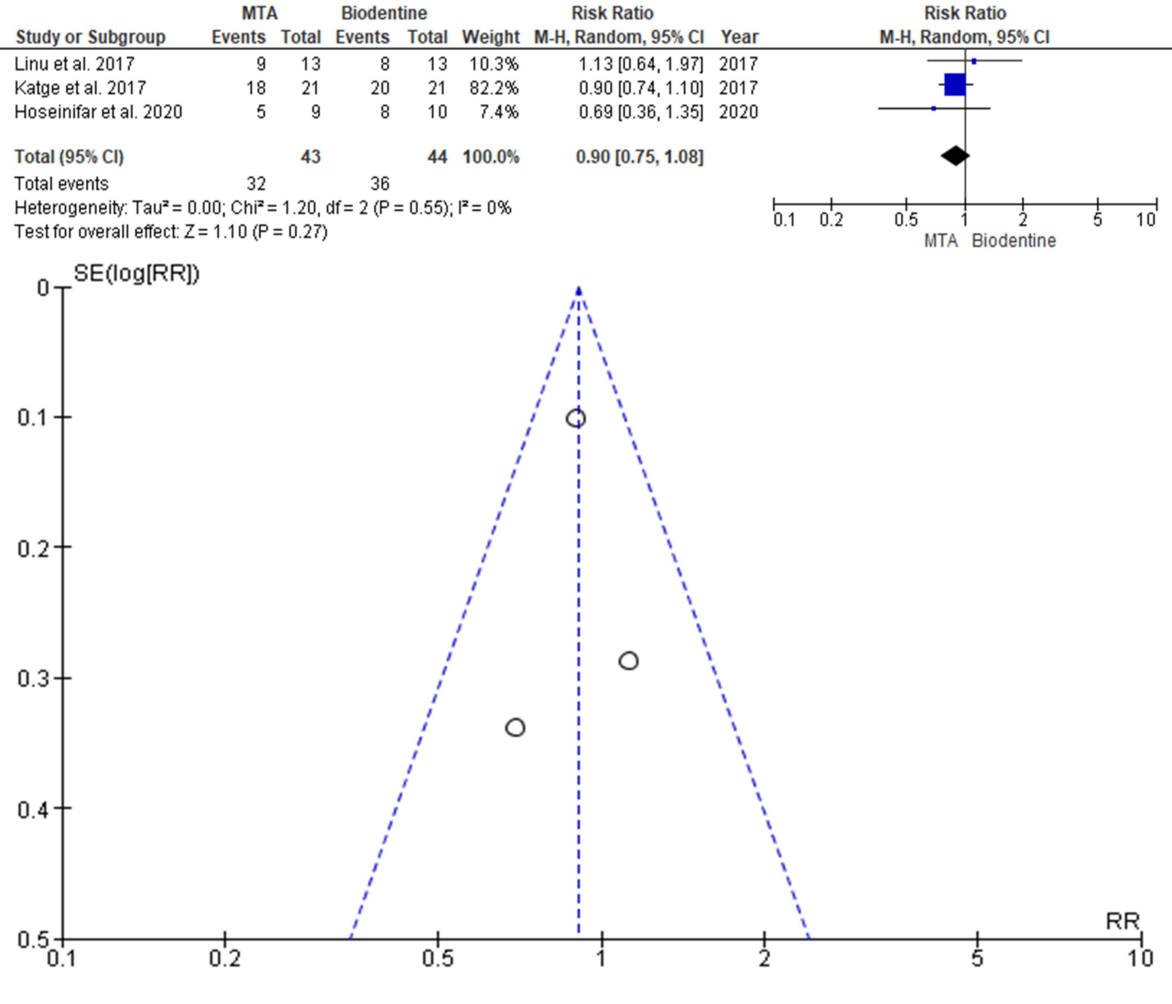
Forest plot showing the comparison between Mineral Trioxide Aggregate and Biodentine with regard to calcific barrier formation and Funnel plot showing asymmetric distribution with absence of systematic heterogeneity Data from [10,11,15].

Data were evaluated from three studies from an aggregate of 87 teeth, of which 43 teeth were evaluated by MTA and 44 teeth were evaluated by BD, for the evaluation of the better efficacy between the two modalities in terms of calcific barrier formation as an outcome. As shown in Figure 5, the risk ratio (RR) is 0.90 (0.75-1.08), and the pooled estimates favor MTA. This signifies that calcific barrier formation on average was 0.90 times more in the MTA group compared to the BD group, and this difference is not statistically significant (p>0.05). The funnel plot did not show significant asymmetry, indicating the absence of publication bias.

Discussion

The findings of the present systematic review underscore the growing global interest in vital pulp therapies, particularly in the use of calcium silicate-based materials such as MTA and BD for DPC in carious mature permanent teeth. The specific selection of mature permanent teeth was made so that the pulp had adequate potential to show responsiveness to the materials [19]. Likewise, the selection criteria employed across the included studies focused on cases with vital pulp, reversible pulpitis, and minimal carious exposure. This uniformity in clinical parameters improves the internal validity of the review. Moreover, the exclusion of advanced pulpal or periapical pathology ensures that the outcomes reflect the true performance of the capping materials rather than the severity of disease. However, variation in patient age across studies, from children to middle-aged adults, may introduce differences in pulpal healing responses. Younger pulps are generally more vascular and cellular, which may enhance reparative capacity compared to those in older adults [19,20].

The widespread geographical distribution of the included studies demonstrates the clinical relevance and acceptance of these materials across various healthcare settings. The consistency in adopting MTA and BD in diverse populations reinforces their versatility and applicability in preserving pulp vitality through biologically based, minimally invasive strategies [21,22]. While MTA has long been considered the benchmark in DPC due to its biocompatibility, sealing ability, and favorable long-term outcomes, it is not without limitations. Its known drawbacks include difficult handling, a long setting time, and the potential for tooth discoloration, especially in esthetically demanding areas, due to the presence of bismuth oxide in white MTA formulations [23,24]. These factors can compromise patient satisfaction and may affect case selection, particularly when anterior teeth or young patients are involved. BD, on the other hand, was designed to overcome many of these drawbacks. Its improved handling properties, shorter setting time, and minimal risk of discoloration make it an appealing alternative, especially in pediatric and esthetic cases [25].

One of the critical clinical advantages of BD noted across the reviewed studies is its dual functionality as both a pulp capping agent and a bulk-filling interim restorative material [26]. This simplifies the operative procedure, reduces material changes during treatment, and shortens chair time, which is beneficial in managing pediatric or anxious patients. The one-step protocol with BD may also reduce the risk of procedural errors and contamination, thereby contributing to favorable outcomes.

Despite differences in clinical handling, both materials demonstrated comparable outcomes in terms of success and survival of treated teeth, suggesting that biological compatibility and the capacity to stimulate dentin bridge formation are primary determinants of success. However, individual studies, such as that by Hoseinifar et al. (2020) [15], have indicated slightly greater dentin bridge thickness in the BD group. This is consistent with in vitro findings that BD releases higher levels of calcium and silicate ions during the early setting phase, potentially accelerating odontoblastic differentiation and mineralization [25]. Nevertheless, the same study also reported increased pulpal inflammation associated with BD, a finding that warrants careful clinical monitoring and further investigation into its long-term implications.

Another notable feature was the consistent use of rubber dam isolation and standard disinfection protocols, such as chlorhexidine and sodium hypochlorite, which play a crucial role in reducing bacterial contamination at the exposure site [27]. These aseptic measures are integral to the success of vital pulp therapies and further support the reliability of the reviewed findings. Most studies used lidocaine with adrenaline, and hemostasis was achieved using sodium hypochlorite or lidocaine-soaked pellets. The effective control of bleeding is paramount before the application of any capping material, as persistent hemorrhage can interfere with the material’s setting and compromise pulp healing. Interestingly, one study achieved hemostasis using only cotton pressure, raising questions about whether chemical agents are always necessary for optimal outcomes [9,28].

Restorative strategies following DPC also varied slightly, with most studies employing resin-modified glass ionomer cement (RMGIC) or composite resin, which are known for their favorable sealing ability and biocompatibility [29,30]. A few studies used amalgam, reflecting either resource constraints or longstanding clinical preferences. Although the restorative material itself was not a primary focus of this review, the ability of the final restoration to maintain a durable coronal seal is critical in preventing microleakage and ensuring the longevity of the pulp capping treatment.

The findings of the meta-analysis further support the narrative synthesis by reinforcing that both MTA and BD exhibit statistically comparable success rates, failure rates, and dentin bridge formation. Although pooled estimates slightly favored MTA in terms of success and calcific barrier formation, and BD in terms of lower failure rate, none of the differences reached statistical significance. These findings confirm that both materials are clinically effective and that their selection can be tailored based on handling preferences, esthetic needs, and cost considerations.

Overall, the findings of the present systematic review and meta-analysis support the notion that both MTA and BD are effective for DPC in carious exposures, with BD offering certain procedural advantages that may make it the material of choice in specific clinical scenarios. Future randomized trials with standardized protocols, larger sample sizes, long-term follow-up, and inclusion of patient-reported outcomes will be essential in refining these findings and guiding evidence-based clinical decision-making.

## Conclusions

Based on the findings of this systematic review and meta-analysis, both MTA and BD are effective direct pulp capping materials for carious exposures in mature permanent teeth, demonstrating high clinical success rates and favorable biological outcomes. While MTA remains a reliable and extensively studied material with excellent sealing and regenerative properties, BD offers distinct clinical advantages, including superior handling, faster setting time, absence of tooth discoloration, and dual functionality as a restorative material. Although the meta-analysis revealed no statistically significant differences between the two materials in terms of success rate, failure rate, or calcific bridge formation, the ease of use and esthetic benefits of BD may render it more suitable in specific clinical scenarios. Therefore, the choice between MTA and BD should be guided by case-specific factors, operator preference, and esthetic considerations, with both materials representing viable options for preserving pulp vitality in minimally invasive endodontic procedures.

## Appendices

The PubMed search strings and filters used in this study are presented in Table 4.

**Table 4:** PubMed search strings and filters used in this study

PubMed Search String:
("Mineral Trioxide Aggregate"[Mesh] OR "MTA" OR "ProRoot MTA" OR "White MTA" OR "Gray MTA") AND ("Biodentine" OR "BD" OR "Calcium Silicate Cement") AND ("Direct Pulp Capping"[Mesh] OR "direct pulp capping" OR "vital pulp therapy" OR "pulp capping") AND ("Permanent Teeth"[Mesh] OR "mature permanent teeth" OR "adult teeth") AND ("Caries"[Mesh] OR "carious exposure" OR "deep caries" OR "dental caries") AND ("Clinical Trial" OR "Randomized Controlled Trial" OR "RCT" OR "retrospective study" OR "comparative study")
Additional Filters:
Language: English
Species: Humans
Publication Dates: From database inception to December 2024
Article types: Clinical Trial, Randomized Controlled Trial, Comparative Study
